# The impacts of implementation of National Essential Medicines Policies on primary healthcare institutions: a cross-sectional study in China

**DOI:** 10.1186/s12913-017-2698-x

**Published:** 2017-11-13

**Authors:** Zhigang Guo, Xiaodong Guan, Luwen Shi

**Affiliations:** 1 School of Pharmaceutical Science, Peking University Health Science Center, 38 Xueyuan Road, Haidian District, Beijing, China; 2 International Research Center of Medicinal Administration, Peking University Health Science Center, 38 Xueyuan Road, Haidian District, Beijing, China

**Keywords:** Essential medicines, Primary healthcare institutions, Health care reform, Drug price markup rate

## Abstract

**Background:**

In 2009, China implemented the National Essential Medicines Policies (NEMPs) as part of a new round of medical system reforms. This study aims to evaluate the impacts of the NEMPs on primary healthcare institutions and discuss the roles of the policies in the new healthcare reforms of China.

**Methods:**

The study selected a total of six representative provinces of China, generating a sample of 261 primary healthcare institutions from August to December in 2010. A questionnaire survey developed by the study team was distributed to all of the primary healthcare institutions. Nine indicators from three dimensions as the outcome variables were used and calculated to evaluate the impacts of implementation of policies. All of the outcome variables were tested using independent-samples T test between the treatment group (with the NEMPs implemented) and the control group (without the NEMPs implemented).

**Results:**

The ratio of drug sales and institution revenues at primary healthcare institutions was 42.99% in the treatment group, which was significantly lower than the control group (53.90%, *p* < 0.01), while the ratio of financial subsidies of the treatment group was shown to be higher (30.78% VS 20.82%, p < 0.01). The rate of healthcare workers income growth was greater in the treatment group (15.35% VS 5.79%, *p* = 0.006). The treatment group exhibited higher outpatient and emergency visits per month in urban areas (2720 VS 1763 visits per month) and rural areas (3830 VS 3633), and higher prescriptions per month in urban areas (2048 VS 1025, *p* = 0.005) and rural areas (3806 VS 3251). The treatment group used more essential medicines and received greater income from essential medicines while the drug price markup rate was lower.

**Conclusions:**

The NEMPs appear to affect the transformation of the operation mechanisms of primary healthcare institutions, the improvement of the mechanisms for government investment, and the healthcare pricing system. Meanwhile, the gaps between urban and rural areas need to be addressed. In conclusion, the NEMPs of China are instrumental to the aim of providing basic healthcare services to every citizen.

## Background

Essential medicines (EMs) are defined by the World Health Organization (WHO) as “those that satisfy the priority health care needs of the population”. In 2015, WHO indicated that over 10 million deaths per year could be avoided by offering health interventions based on the delivery of EMs [1]. Essential medicines policies (EMPs) can help countries to rationalize the purchasing and distribution of medicines, thereby reducing costs to public and private health systems, improve the accessibility of medicines and enhance the rational use of medicines [2–4].

In 1979, China adopted the concept of EMs yet it had limited impacts on its healthcare system until 2009 [2]. In March 2009, the Chinese government released the first detailed 3-year implementation plan that was designed to deepen healthcare reform [5]. This reform explicitly set “Preliminarily establishment of a national essential medicines system” as one of the top five priorities in order to increase the accessibility of cost-effective medicines and decrease drug costs [6–8]. The main policies of the National Essential Medicines Policies (NEMPs) included [9–12]: (1) Establishing a system for the selection, production and supply as well as the use and reimbursement of EMs; (2) The introduction of an essential medicines list (EML) that specifies which medicines can be used in all of the government-sponsored primary healthcare institutions and the prohibition of profit (the “zero mark-up” policy) [13]. Each province can add provincial supplement essential medicines (PSEMs) to the EML to meet different potential clinical requirements [2, 5]; (3) Efforts should be made to improve the reimbursement policies concerning the EMs. The reimbursement rate of the EMs, including the PSEMs, should be significantly higher than non-essential medicines (non-EMs).

Since 2009 many studies on the impacts of NEMPs in China have demonstrated the effects of the policies in promoting the availability and the affordability of medicines [14], decreasing drug prices [7, 15] and how to improve the rational use of medicine [16, 17]. However, few studies have presented the NEMPs’ roles in the reforms since their prioritization in 2009. The Chinese government expected the NEMPs to assist in the transformation of operation mechanisms, improve government investment mechanisms, and establish a sound healthcare pricing system in grass-roots primary healthcare institutions [2, 9, 10]. Based on these factors, the Chinese government aims to strengthen the service capabilities of government-sponsored primary healthcare institutions, change profit-driven behaviour and gradually achieve the goal that every citizen is entitled to basic healthcare services [18–20].

Few previous studies have examined the related results. Prior studies respectively focused on the role of the NEMPs in increasing the utilization [21], the proportion of total sales of EMs [22], the increased financial subsidies which made healthcare institutions rely more on public financing subsidies [23], the increased use of injections [24], changing the profit-seeking behaviours of healthcare workers [25], among other aspects. Given the lack of uniformity and inconclusive results of previous studies, comprehensive research that focuses on the overall impacts of the policies on all aspects of primary healthcare institutions in China is necessary.

Our study aims to comprehensively evaluate the impacts of the NEMPs on primary healthcare institutions and discuss the roles of the policies in the recent healthcare reform in China. We focused on the impacts on the income structure, the operating situation, and the drug purchasing and selling of primary healthcare institutions in order to explicate the various action mechanisms.

## Methods

### Sampling

In 2009, the NEMPs were required to be implemented in at least 30% of all primary healthcare institutions of every province in China [26, 27]. The following year we conducted a cross-sectional survey and selected a total of six representative provinces in eastern (Beijing, Shanghai, Tianjin), central (Anhui, Hubei), and western (Yunnan) of China. Then we randomly selected 12 primary healthcare institutions in rural and urban areas respectively for the treatment group (with the NEMPs implemented) and control group (without the NEMPs implemented) separately in each province, resulting in 288 institutions (48 for each province).

### Questionnaire design and data collection

In August 2010, a questionnaire developed by the study team was distributed to all 288 institutions. Table 1 shows the structure and contents of the questionnaire. At the end of December 2010, 261 institutions from six provinces returned the questionnaire. The valid response rate of the questionnaires was 90.6% (261/288). However, only 229 institutions completed Part A and Part C, and 201 institutions returned Part B. Because Beijing, Tianjin, Anhui and Shanghai lacked data in the control and treatment groups in Part B, our analysis included only Hubei and Yunnan as samples (71 institutions).

**Table 1:** The structure and contents of the surveyed questionnaire

Items	Indicators	Time frame
Part A The Incomes	the total revenues of the institution (Yuan) (A1)	From 1 January 2010 to 30 June 2010
	the total revenues of the outpatient (Yuan) (A2)	
	the total financial subsidies (Yuan) (A3)	
	the total sales of the drugs (Yuan) (A4)	
	the total sales of the drugs of the outpatient (Yuan) (A5)	
	the rate of healthcare workers income growth^a^ (A6)	From 1 January 2009 to 30 June 2010
Part B The Visits and Prescriptions	the average outpatient and emergency visits per month (B1)	From 1 January 2010 to 30 June 2010
	the average prescriptions per month (B2)	
Part C The Medicines^b^	the number of EMs (C1)	
	the number of PSEMs (C2)	
	the number of non-EMs (C3)	
	the sales of EMs (Yuan) (C4)	
	the sales of PSEMs (Yuan) (C5)	
	the sales of non-EMs (Yuan) (C6)	
	the average price markup rate^c^ of EMs (C7)	
	the average price markup rate of PSEMs (C8)	
	the average price markup rate of non-EMs (C9)	
	the average price markup rate of all medicines (C10)	

^a^The rate of healthcare workers income growth $=\Big(\frac{\mathrm{theaverageincomespermonthin}2010}{\mathrm{theaverageincomespermonthin}2009}$ – 1)*100%

^b^The EMs were medicines on the national essential medicines list (a total of 307 drugs) by the central government, and the PSEMs were medicines added into the list by each province (Shanghai added 410 drugs, Tianjin added 230, Anhui added 305, Hubei added 177, Yunnan added 169 and Beijing had not added). Other medicines were the non-EMs

^C^The average price markup rate of an institution was the mean value of the price markup rate of every medicine, and the price markup rate of a medicine $={\frac{\mathrm{thesaleprice}-\mathrm{thepurchaseprice}}{\mathrm{thepurchaseprice}}}^{\ast }100\%$

### Measurements

The indicators we employed to evaluate the impacts of the implementation of NEMPs included:

1. The institution income structure: the ratio of outpatient drug sales of outpatient revenues (A5/A2), the ratio of drug sales of institution revenues (A4/A1), the ratio of financial subsidies of institution revenues (A3/A1), and the rate of healthcare workers income growth (A6) were chosen to represent the institution income structure.
2. The operating situation of institutions: the average outpatient and emergency visits per month (B1) and the prescriptions per month (B2) were selected to represent the operating situation of institutions.
3. Drug purchasing and sales at institutions: the number ratio of EMs of all medicines ($\frac{\mathrm{C}1}{\mathrm{C}1+\mathrm{C}2+\mathrm{C}3}$), PSEMs of all medicines $\left(\frac{\mathrm{C}2}{\mathrm{C}1+\mathrm{C}2+\mathrm{C}3}\right)$ and non-EMs of all medicines $\left(\frac{\mathrm{C}3}{\mathrm{C}1+\mathrm{C}2+\mathrm{C}3}\right)$ were employed to represent drug purchasing. The sales ratio of EMs of all medicines $\Big(\frac{\mathrm{C}4}{\mathrm{C}4+\mathrm{C}5+\mathrm{C}6}$), PSEMs of all medicines $\Big(\frac{\mathrm{C}5}{\mathrm{C}4+\mathrm{C}5+\mathrm{C}6}$), non-EMs of all medicines $\left(\frac{\mathrm{C}6}{\mathrm{C}4+\mathrm{C}5+\mathrm{C}6}\right)$ and the average price markup rate of EMs (C7), PSEMs (C8), non-EMs (C9) to represent the sales. Concurrently, with C7, C8, C9, C10, we could evaluate the influences on the health care pricing system.

### Statistical analysis

The research team outlined the means (normally distributed data) of all the outcome variables described above, both in the treatment group and the control group. These means reflect the differences between the two groups and thus can be used to evaluate the impacts of implementation of NEMPs. The results of the differences were tested using independent-samples T test.

## Results

### The impact on the income structure of institutions

Table 2 shows that the ratio of outpatient drug sales of outpatient revenues in the treatment group was lower (66.00% VS 70.78%, *p* = 0.009) than control group. The same result appeared in the analysis in urban areas (67.29% VS 72.39%), but the opposite result occurred in rural areas (64.79% VS 64.13%).

**Table 2:** The outcomes of the institution income structure

	total		P	urban		P	rural		P
	The treatment group (*n* = 142)	The control group (*n* = 87)		The treatment group (*n* = 68)	The control group (*n* = 70)		The treatment group (*n* = 74)	The control group (*n* = 17)	
1 Outpatient drug sales/outpatient revenues	66.00%	70.78%	0.009	67.29%	72.39%	0.055	64.79%	64.13%	0.555
2 Drug sales/institution revenues	42.99%	53.90%	0.000	44.06%	56.71%	0,000	42.05%	42.34%	0.471
3 Financial subsidies/institution revenues	30.78%	20.82%	0.000	32.13%	18.60%	0.000	29.57%	29.38%	0.390
4 Rate of medical workers incomes growth	15.35%	5.79%	0.006	19.90%	5.96%	0.008	11.82%	2.01%	0.225

The ratio of drug sales of institution revenues was 42.99% in the treatment group, which is significantly lower than the value in the control group (53.90%, *p* < 0.01). In the separate analysis in urban areas (44.06% VS 56.71%, *p* < 0.01) and rural areas (42.05% VS 42.34%), the results showed the same trend. On the other hand, the ratio of financial subsidies of institution revenues was higher in the treatment group than the control group (30.78% VS 20.82%, *p* < 0.01), and this was consistent in urban areas (32.13% VS 18.60%, p < 0.01) and rural areas (29.57% VS 29.38%).

The healthcare workers’ incomes in treatment group and control group in both the urban areas and rural areas increased when the growth rate was greater in treatment group (15.35% VS 5.79%, *p* = 0.006). The growth rate in urban areas was 3-4 times greater than that of the control group (19.90% VS 5.96%, *p* = 0.008) and in rural areas was 5-6 times greater.

### The impact of the operating situation of institutions

The treatment group exhibited higher outpatient and emergency visits per month (Table 3) in urban areas (2720 VS 1763 visits per month) and rural areas (3830 VS 3633), which is not statistically significant. Meanwhile, the treatment group and the control group in rural areas both showed higher numerical values than in urban areas (3830 VS 2720 and 3633 VS 1763).

**Table 3:** The outcomes of the operating situation of institutions

	urban		P	rural		P
	The treatment group (*n* = 18)	The control group (*n* = 13)		The treatment group (*n* = 29)	The control group (*n* = 11)	
1 The average outpatient and emergency visits per month	2720 (2085)	1763 (1431)	0.183	3830 (3045)	3633 (3184)	0.858
2 The average prescriptions per month	2048 (1469)	1025 (752)	0.005	3806 (3290)	3251 (2907)	0.728

For the number of prescriptions (Table 3), the treatment group had higher prescriptions per month in both urban areas and rural areas. In urban areas, the number of prescriptions per month reached 2048 prescriptions in the treatment group, which is significantly higher than those in the control group (1025 prescriptions, *p* = 0.005). The number of prescriptions per month of the treatment group and the control group in rural areas was higher than in urban areas (3806 VS 2048 and 3251 VS 1025 prescriptions).

### The impact on drug purchasing and sales of institution

The number ratio of EMs in the treatment group was higher in urban areas (55.65% VS 40.24%, *p* = 0.001) while the number ratio of PSEMs was less (23.45% VS 35.85%, p = 0.001), and the number ratio of non-EMs was almost the same (20.90% VS 23.92%). However, the result in rural areas was the opposite in the number ratio of EMs (65.10% VS 73.11%) and PSEMs (19.70% VS 11.76%). The number ratio of non-EMs in rural areas was also approximately the same (15.20% VS 15.12%).

The sales ratio of EMs of all medicines in the treatment group was higher in urban areas (48.88% VS 27.23%, *p* = 0.001) while the sales ratio of PSEMs was not (25.41% VS 49.10%, *p* < 0.01), and the sales ratio of non-EMs was similar (25.71% VS 23.67%). In rural areas the results of EMs (60.22% VS 72.86%) and PSEMs (21.37% VS 9.03%, *p* = 0.009) was opposite to the urban areas between the treatment group and the control group. In both urban areas and rural areas, the drug sales of the institutions mainly derived from EMs.

The average drug price markup rate of EMs and PSEMs in treatment group was very low in urban areas (0.28% and 0%) and rural areas (0.46% and 0.05%). The average drug price markup rate of EMs and PSEMs in the control group was close to 15% in urban areas (15% and 15.12%) and rural areas (14.82% and 14.36%). This different markup rate of EMs or PSEMs in the treatment and the control group was statistically significant. The average drug price markup rate of non-EMs in the treatment group was lower in urban areas (10.21% VS 15%, *p* = 0.003) and rural areas (7.91% VS 10%, *p* = 0.004). The average markup rate of overall drugs in the treatment group was significantly lower in urban areas (2.32% VS 14.98%, *p* = 0.001) and rural areas (2.66% VS 13.40%, p = 0.003).

## Discussion

The NEMPs may be associated with the transformation of the operation mechanisms of primary healthcare institutions and improving the mechanisms for government investment, which is similar to a previous research [28]. The ratio of outpatient drug sales of outpatient revenues and the ratio of drug sales of institution revenues were lower in the treatment group which implied that the primary healthcare institutions reduced their dependence on drug income. Moreover, the higher rate of healthcare providers’ income growth also implied this phenomenon. On the other hand, the financial subsidies increased in the treatment group, and the sum of the ratio of the drug sales and the financial subsidies of the income of primary healthcare institutions were similar between the two groups, showing that government investments appear to promote the transformation of the operation mechanism, in accordance with other studies’ findings [25, 29]. Further research also demonstrated that the income of primary healthcare providers derived mainly from the medical service charges and government subsidies [24, 30], if the institution had implemented the NEMPs.

Additionally, the NEMPs, among other factors, had a relationship with the utilisation of primary healthcare services. Other studies had showed the weaknesses of the primary healthcare were one of the most important reasons to the difficulties and high expenses in medical care [31–33]. Because the NEMPs played a part in the supply of medicines and decreasing the drug price makeup rate in primary healthcare institutions and the reimbursement rate of the EMs was significantly higher, more patients would like to return to primary healthcare. Therefore, the average number of outpatient and emergency visits per month was higher in the treatment group. Moreover, the average number of prescriptions per month was also higher. The more visits, the more prescriptions, and the higher rate of healthcare providers’ income growth were beneficial to strengthen the service capabilities of the primary healthcare institutions. However, one research indicated that [34], it is worth considering that in China the NEMPs focus on strengthening the utilisation of medicines and lack the specific measures on training healthcare workers, and improving medical technologies, which were equally important in the institutional framework of EMPs set out by the WHO [35].

The NEMPs seemingly decrease the level of the drug price and affect the health care pricing system. Because the drug markup policy required the markup rate could not exceed 15%, which was instated to compensate for hospital costs [36], the average markup rate of three kinds of medicines in the control group was close to 15%. This policy was regarded as an important contributing factor to the unreasonable price system and the high drug expenditures [36, 37]. However, the average markup rate of the treatment group approached 0%. This also contributed to reducing the dependence on the drug income of the institutions.

We found that approximately four-fifths of medicines used in the primary healthcare institutions were the EMs and PSEMs in both groups. The sales from EMs and PSEMs occupied the greater parts of the drug sales. That is to say, the majority of healthcare providers might have chosen to take advantage of EMs and PSEMs at primary healthcare institutions where the treatment group used more EMs. Therefore, the selection of the EMs and PSEMs was a decisive factor in deciding whether the primary healthcare institution could meet needs of the patients and whether the policies could act effectively, as identified by previous studies [38, 39]. The impacts of the selection would expand when the drug price markup rate of medicines was significantly reduced, which in turn made the policies more attractive.

Finally, one major obstacle might be the gaps in the implementation of the NEMPs between urban areas and rural areas. In Table 4, the institutions and the patients of rural areas relied more on the EMs and PSEMs. At the same time, the goal of NEMPs was also to ensure the equity in access to the most basic drugs and their rational use. Nonetheless, the results in our study showed almost no differences between two groups in the rural areas. This implied that the implementation of NEMPs may have been obstructed in rural areas, when several studies attributed the obstruction to the deficiency [40] and inequity [41–43] of financial subsidies. Also, Table 2 shows the financial subsidies did not change after the introduction of NEMPs in the rural areas, resulting in the amount ratio and the income ratio of EMs did not change (Table 4). Thus the average outpatient and emergency room visits per month and the average prescriptions per month in institutions of the rural areas did not change (Table 3). These gaps or inequities between urban areas and rural areas were regarded as the potential in constraining the advancement of the NEMPs by others’ research results [21, 44].

**Table 4:** The outcomes of drug purchasing and sales of institution

	urban		P	rural		P
	The treatment group (*n* = 68)	The control group (*n* = 70)		The treatment group (*n* = 74)	The control group (*n* = 17)	
1 The ratio of the number of						
-EMs/all medicines	55.65%	40.24%	0.001	65.10%	73.11%	0.367
-PSEMs/all medicines	23.45%	35.85%	0.001	19.70%	11.76%	0.160
-non-EMs/all medicines	20.90%	23.92%	0.337	15.20%	15.12%	0.991
2 The sales ratio of						
-EMs/all medicines	48.88%	27.23%	0.001	60.22%	72.86%	0.272
-PSEMs/all medicines	25.41%	49.10%	0.000	21.37%	9.03%	0.009
-non-EMs/all medicines	25.71%	23.67%	0.605	18.42%	18.11%	0.973
3 The average price markup rate of						
-EMs	0.28%	15.00%	0.001	0.46%	15.12%	0.009
-PSEMs	0.00%	14.82%	0.000	0.05%	14.36%	0.001
-non-EMs	10.21%	15.00%	0.003	7.91%	10.00%	0.004
-all medicines	2.32%	14.98%	0.001	2.66%	13.40%	0.003

### Limitations

This study could not compare the impacts of pre- and post-implementation of NEMPs on institutions, with a non-randomized controlled design and the absence of evidences in the characteristics of institutions, so we could not conclude a causal relationship between the policies and the outcome indicators. Moreover, because of the lack of continuous observation on the policies, the effects of the NEMPs on institutions may be underestimated. Further studies are warranted and more empirical data are needed.

## Conclusion

The NEMPs of China are shown to have a relationship with transforming the operation mechanism of primary healthcare institutions and improving the mechanism for government investment while these increase the burden of the financial subsidies. The policies also appear to affect the utilisation of the primary healthcare and decrease the level of the drug price, thus improving the healthcare pricing system. Basing on these, the selection of EMs and PSEMs would play the important roles. The next stage of NEMPs should focus on the elimination of inequities between urban and rural areas across China.

## Abbreviations

EML: Essential Medicines List
EMPs: Essential Medicines Policies
EMs: Essential Medicines
NEMPs: National Essential Medicines Policies
non-EMs: Non-Essential Medicines
PSEMs: Provincial Supplement Essential Medicines
WHO: World Health Organization
